# Benefits & Barriers: Improving Medical Handover in a Psychiatric Hospital

**DOI:** 10.1192/bjo.2024.391

**Published:** 2024-08-01

**Authors:** Nina MacKenzie, Callum Cruickshank, Ewan Mahony, Jessica Parker, Robyn Canham

**Affiliations:** 1 NHS Education Scotland, Edinburgh, United Kingdom; 2 NHS Lothian, Edinburgh, United Kingdom

## Abstract

**Aims:**

*Background:* Handover aims to achieve the efficient communication of clinical information when responsibility for patients is transferred. The Royal Edinburgh Hospital (REH), a specialist hospital serving the Lothians, has repeatedly received “red flags” (ranked in the bottom 2% of benchmarked areas) on the handover section of the Scottish training survey (STS) and GMC national training survey of doctors in training (DiT).

*Aims:*

- Survey DiT to understand their experience of handover.
- Introduce a new structured handover process.
- Re-audit parameters after intervention.

**Methods:**

Data from REH DiT were extracted from an anonymised handover survey, disseminated to all psychiatry DiT in Scotland in January 2023. Multiple choice and free-text questions covered handover timings, format, structure, and attendance. The survey was repeated after intervention. In addition, data from the STS were analysed. The intervention consisted of altering shift times to include protected time for handover, introducing a dedicated room, training in the use of an electronic system to record tasks, involvement of senior doctors, and dissemination of the new changes to procedure.

**Results:**

A total of 12 survey responses (25% response rate) pre-intervention (25% FY2s, 17% GPSTs, 58% core trainees) and 14 post-intervention (14% FY2s, 14% GPSTs, 71% core trainees) were analysed. The proportion of respondents reporting that handover always happened at times of shift change increased from 7% to 93% post-intervention. The proportion of those reporting that there was protected time for handover rose from 0% to 50%, and the use of a predetermined structure/format increased from 0% to 43%. After intervention, 86% of DiT felt adequately supported during handover (compared with 17% pre-intervention) and 93% of respondents felt handover ‘allowed for the efficient and effective transfer of information to protect patient safety’ (33% pre-intervention). Prior to the process change, 83% of DiT felt there was no clear senior leadership at handover; this fell to 21%. Post-intervention the use of WhatsApp/texts to hand over information fell by 100%. The new system was welcomed by trainees, but teething problems were identified.

**Conclusion:**

The new process led to improvements in the frequency, consistency, format, recording, and senior support of handover. Issues with the use of video call software and electronic medical records systems have been identified, and work is ongoing to address these in an iterative quality improvement process. Good clinical handover benefits patients (fewer mistakes and increased safety, better continuity of care, improved satisfaction) and clinicians (improved communication skills, increased accountability, feel more informed, improved job satisfaction).

